# The effect of eye care protocol on the prevention of ocular surface disorders in patients admitted to intensive care unit

**DOI:** 10.25122/jml-2022-0139

**Published:** 2022-08

**Authors:** Fariba Mobarez, Neda Sayadi, Simin Jahani, Asaad Sharhani, Mohsen Savaie, Fereydoun Farrahi

**Affiliations:** 1 Nursing Care Research Centre in Chronic Diseases, Ahvaz Jundishapur University of Medical Sciences, Ahvaz, Iran; 2 Department of Medical and Surgical Nursing, School of Nursing and Midwifery, Nursing Care Research Centre in Chronic Diseases, Ahvaz Jundishapur University of Medical Sciences, Ahvaz, Iran; 3 Department of Biostatics and Epidemiology, School of Health, Ahvaz Jundishapur University of Medical Sciences, Ahvaz, Iran; 4 Department of Anesthesiology, School of Medicine, Pain Research Center, Ahvaz Jundishapur University of Medical Sciences, Ahvaz, Iran; 5 Department of Ophthalmology, School of Medicine, Infectious Ophthalmologic Research Center, Ahvaz Jundishapur University of Medical Sciences, Ahvaz, Iran

**Keywords:** eye care, dry eye syndrome, infectious keratitis, conjunctivitis, intensive care unit

## Abstract

Eye care is one of the most critical tasks of intensive care unit (ICU) nurses. Patients in this unit are exposed to potential ocular problems due to critical conditions. This study aimed to establish a new eye care protocol for preventing ocular surface disorders in patients admitted to ICU. This was a clinical trial study performed on patients admitted to ICU in 2019. The data gathering tools included the demographic questionnaire, the Schirmer test for dry eye, fluorescein staining and slit lamp manual for examining corneal ulcers, and slit lamp manual to check keratitis and conjunctivitis. A type of eye care protocol was performed on the patient's eyes. After five consecutive days of executing the protocol, the data were analyzed using SPSS software version 18. The use of eye care protocol reduced the risk of keratitis (P=0.027), conjunctivitis (P=0.012), eye dryness (P=0.001), and corneal ulcer (P=0.003) in patients admitted to ICU in the intervention group compared to the control group. Ophthalmology protocols reduced the incidence of keratitis, conjunctivitis, dry eye, and corneal ulcers in ICU patients. Therefore, using this method in ICU patients can improve nursing care.

## INTRODUCTION

In healthy people, tears act as a mechanical lubricant, keeping the eye's surface moist and destroying microorganisms with their antimicrobial properties. In addition, the eyelids act as a physical barrier to prevent strike and dry eyes [1]. A number of hospitalized patients in intensive care units (ICU) are at greater risk due to defects in the eye's defense mechanisms. This is because defects in the eye's protective mechanisms, especially in patients with decreased levels of consciousness and in patients receiving sedatives and neuromuscular relaxants, cause ocular surface disorders [2]. On the other hand, due to incomplete closure of the eyelids, tears evaporate much faster, and the use of drugs such as antihistamines, atropine, phenothiazines, and tricyclic antidepressants exacerbate this complication [3]. These factors cause serious damage to the defense surface of the conjunctiva and cornea. However, another problem is that patients admitted to special wards are not evaluated in time due to limitations in expressing their eye complaints, receiving delayed diagnosis and treatment, and leading to serious injury to a person's vision [4].

Eye injuries in Iran can be as mild as infection or as severe as corneal rupture and can even cause permanent damage and loss of vision. According to recent research, the rate of corneal injuries in hospitalized patients in intensive care units was 59.4% [2]. Also, eye disorders that commonly occur in the intensive care unit can cause keratopathy (3–60%), comorbidity (9–80%), and microbial keratitis, and in case of improper care, can eventually lead to blindness [5]. Common eye complications in the intensive care unit include dry eye, keratitis, conjunctivitis, and corneal abrasions due to infection and inflammation caused by the virus. Microbial keratitis is a potential factor contributing to infection and destruction of the cornea. Corneal abrasion is a traumatic defect in the corneal epithelium that can be caused by mechanical trauma due to the use of a suction catheter, tubes attached to the patient, and a change in the patient's position [5].

Eye care is one of the most critical tasks of nurses in the intensive care unit; however, due to the critical situation and many treatment procedures done for patients admitted to the ICU, eye care could be done improperly. As the focus is on treating the main organs, eye care is considered a side issue [6]. There have been many studies on eye care in ICU, including washing with sterile water and normal saline every 2–4 hours, using a polyethylene coating, eye ointment, tetracycline ointment, gentamicin ointment, lubricant liquid, methylcellulose ointment, paraffin gas, swimming goggles, artificial tear drops or closing the eyes [7]. Most studies examined the effect of one or two comparisons, and the implementation of eye protocols and accurate evaluation was considered. In patients in the intensive care unit who are unconscious or have a reduced level of consciousness and are under mechanical ventilation, implementing eye care protocols is essential and reduces the incidence of eye complications and prevents ocular surface disorders [6]. Kousha *et al*. (2014) found that using a simple eye protocol could reduce the incidence of keratopathy in adults admitted to the ICU and exposed to critical keratopathy [8].

Despite sporadic intervention studies in Iran, but probably because of the lack of proper and appropriate tools, eye care is not adequately performed; therefore, the rate of eye problems, including eye infections, is still high [9]. Evaluation, intervention, and eye care methods are different in eye care protocols, and there are no systematic and structured registration forms for evaluation. Therefore, it can lead to errors in reporting or reduced awareness of how to implement protocols [10]. Due to the lack of special protocols for eye care in patients hospitalized in special wards and the lack of review of various methods of eye care, employees choose one of the methods as per their personal opinion and act accordingly [11]. Recently, guidelines for eye care procedures have been developed in special wards that recommend the use of a simple eye protocol and good nursing to prevent eye complications [10]. Neglecting eye care in the ICU can have serious physical, mental, financial, and economic costs for the patient [12]. Reviews of various studies showed that there are few protocols for eye care in hospitalized patients in special wards, the effect of different methods has not been properly determined, and clinical trials have found different findings. Therefore, the aim of this study was to determine a new protocol for eye care to prevent ocular surface disorders in hospitalized patients in the intensive care unit.

## Material and Methods

This was a clinical trial study (#IRCT20190522043671N1) performed on patients admitted to the intensive care units of Imam Khomeini and Golestan Hospital at Ahvaz in 2019. Participants were selected from a research community who were eligible to participate. They were assigned to intervention and control groups randomly. The allocation of the type of intervention to the subjects in the study (nurses) was random and by the method of random blocks with block size 4 (using the table related to random replacements). A random list was prepared by a statistician. The type of intervention was assigned to each person who entered the study according to a random list and the corresponding codes. Inclusion criteria were age over 18 years and under 75, level of consciousness less than 8, patients with mechanical ventilation and impaired eyelid reflexes, no history of hospitalization in the intensive care unit for the last month, no history of eye problems (eye diseases), lack of use of ophthalmic medications such as corticosteroid eye drops, no allergy to eye lubricants, no eye trauma, no symptoms of increased intracranial pressure, healthy corneal surface at the initial examination and at least 24 hours after ICU hospitalization. Exit criteria were the return of the blinking reflex during the study period, discharge or transfer from the intensive care unit, and death before the end of the study period.

Data collection tools in this study consisted of 4 sections. The first section included a demographic questionnaire that identified age, sex, hospitalization time, history of care in the intensive care unit over the past month, diagnosis, and use of suction. The second part was the use of Schirmer strips, which are used to check for dry eye, and the third part was the use of fluorescein staining and hand-held and portable slit lamps, which are used to examine corneal ulcers [9]. The fourth part included a hand-held slit lamp device used to examine keratitis and conjunctivitis in hospitalized patients in the intensive care unit [10]. Thus, after observing the symptoms of conjunctivitis, including red conjunctivitis with watery and swollen eyelids, and the symptoms of keratitis, such as white or yellow spots on the surface of the cornea, a lamp slit device was used for the final diagnosis [12]. According to previous studies in Iran, fluorescein staining and the Schirmer test have a reliability of 84% and a total correlation of 90% [5, 13]. In this study, after conscious consent of the patient's family, a type of eye care protocol was performed for the patient to determine the incidence of eye complications after using this protocol. Initially, the protocol implementation method was taught to ICU nurses by an ophthalmologist and the researcher before the intervention. The ophthalmologist also evaluated the patients, and qualified patients entered the study after 24 hours of hospitalization in the intensive care unit. During the initial examination, the health of the cornea in eligible patients was examined using fluorescein staining and the Schirmer test. The Schirmer test was used to check for dry eye by placing it on the edge of the eyelid; if the strip was wet to the line of 15 ml, it indicated that the eye was not dry. Fluorescein staining and hand-held and portable slit lamp were also used to examine corneal ulcers. We moistened the sterile paper strips containing fluorescein (yellow) and placed them on the inner surface of the lower eyelid temporarily to dissolve the color in tears, and then the cornea was examined by an ophthalmologist with the blue light of a slit lamp. Normally, a thin, uniform layer (blue) was placed on the cornea. If the surface of the cornea was abnormal, the color would accumulate there; a non-uniform area (green) could be observed compared to other areas of the cornea [10].

The study looked at the effect of eye care, including keeping the eye closed with adhesive tape in patients whose eyelids were incompletely closed, as well as keeping the eye moist with 0.3% hypromellose tear drops on ocular surface disorders. The eye disorders were keratitis, conjunctivitis, corneal ulcers, and dry eyes. This care was performed on both eyes in the intervention group and compared with patients' eyes in the control group.

Eye care protocol in eligible patients hospitalized in the ICU: in the first stage, the position of the eyelids was evaluated, and according to this assessment, patients were divided into three groups, and appropriate eye care was performed in each group.

1. The first group consisted of patients with closed eyelids. In this group, one drop of hypromellose 0.3% was applied to both eyes every 2 hours and continued for 4 hours.
2. In the second group, only the conjunctiva was visible. In this group, in addition to using three drops of hypromellose 0.3% every 2 hours that lasted for 6 hours, the adhesive tape was also used horizontally across the eye to keep the eyes closed (a piece of adhesive was used horizontally on the upper eyelid and another adhesive under the lower eyelid and the skin of the face).
3. In the third group, the patient's cornea was exposed and these patients were at greater risk. In this group, three drops of hypromellose 0.3% were applied every 2 hours (for 8 hours), and the adhesive tape was used horizontally throughout the eye to keep the eyes closed.

Eye care and its evaluation were performed by trained nurses on each shift. First, patients' eyes were washed with saline, and then eye care was provided. It should be noted that all patients in the control group received routine eye care every 2 hours (including washing the eyelid and surrounding skin using gas and sterile water). This eye care was performed for 5 days, and the ophthalmologist, without knowing the method of eye care, evaluated the patients before and after the intervention using fluorescein staining, Schirmer test, and slit lamp in terms of ocular surface disorders. The final evaluation was then performed by an ophthalmologist using a portable slit lamp, as well as the nurses' daily notes, ICU charts, and the occurrence of keratitis and conjunctivitis. The presence of white or yellow spots in the cornea was a sign of keratitis, and red conjunctiva with tearing and swelling of the eyelids indicated that conjunctivitis would develop. Patients with ocular surface disorders were treated by an ophthalmologist or ICU physician.

Data analysis was conducted by SPSS 18 statistical software using the Chi-square test, independent t-test, and odds ratios. Significant levels were considered below 0.05.

## Results

In the control group, two participants were dropped from the sample. One participant was excluded due to death and the other due to complete recovery of consciousness.

According to Table 1, the demographic and clinical variables, including Glasgow Coma Scale (GCS)(P=0.609), age (P=0.618), hospitalization time (P=0.780), the rate of suction use (P=0.363), gender (P=0.843), type of intervention (P=0.208) and type of diagnosis (P=0.923) did not show a statistically significant difference between the intervention and control groups.

**Table 1:** Comparison of demographic characteristics between intervention and control groups among hospitalized patients at ICU.

Variable		Control group (n=32)	Intervention Group (n=34)	P-value
		SD±Mean	SD±Mean	
**GCS**		1.41±6.25	1.18±6.41	0.609
**Age**		14.25±52.56	16.74±50.50	0.618
**Duration of hospitalization**		29.22±60.75	31.82±62.82	0.780
**The amount of suction used**		0.65±0.97	0.69±1.12	0.363
		**Number (percentage)**	**Number (percentage)**	
**Gender**	Male	13 (40.6)	13 (38.2)	0.843
	Female	19 (59.4)	21 (61.8)	
**Type of intervention**	Closed eyelids	4 (12.5)	10 (29.4)	0.208
	Half-closed eyelids	19 (59.4)	18 (52.9)	
	Open eyelids	9 (28.1)	6 (17.6)	
**Diagnosis**	Trauma	10 (31.1)	22 (68.8)	0.923
	No trauma	11 (32.4)	23 (67.6)	

According to Table 2, the incidence of keratitis in the control and the intervention group was significantly different (P=0.027, 95% CI: 1.12–8.85, OR=3.1). Also, the occurrence of conjunctivitis in the control and intervention groups were significantly different (P=0.012, 95% CI: 1.30–10.20, OR=3.7). Furthermore, the incidence of dry eye in the control and the intervention group was significantly different (P=0.001, 95% CI: 1.85–15.15, OR=5.3). There was also a significant difference between the occurrence of corneal ulcers in the control and the intervention group (P=0.003, 95% CI: 1.65–13.33, OR=4.8).

**Table 2:** Comparison of keratitis, conjunctivitis, dry eyes, and corneal ulcers after the intervention between groups.

Variable		Control group (n=32)	Intervention group (n=34)	OR*	X^2^	P-value	95% CI
		Number (percentage)	Number (percentage)				
**Keratitis**	Positive	17 (53.1)	9 (26.5)	3.1	4.91	0.027	1.12-8.85
	Negative	15 (46.9)	25 (73.5)				
**Conjunctivitis**	Positive	23 (71.9)	14 (41.2)	3.7	6.31	0.012	1.30-10.20
	Negative	19 (28.1)	20 (58.8)				
**Eye dryness**	Positive	22 (68.8)	10 (29.4)	5.3	10.21	0.001	1.85-15.15
	Negative	10 (31.3)	24 (70.6)				
**Corneal ulcer**	Positive	23 (71.9)	12 (53.3)	4.8	8.86	0.003	1.65-13.33
	Negative	9 (28.1)	22 (64.7)				

* – The ratio of the control group's chance to the intervention group.

## Discussion

The present study showed that using eye care protocol reduced the incidence of keratitis in hospitalized patients in special wards in the intervention group compared to the control group. In the study of Kousha *et al*., eye ointment in patients under mechanical ventilation and sterile eye gas in conscious patients were used daily, and their eyelids were cleaned. In the first stage, the incidence of keratopathy was generally 21%, with 56% reported in patients undergoing mechanical ventilation. In the second stage and after the protocol implementation, the incidence of keratopathy decreased. Finally, using a simple eye care protocol can reduce the prevalence of this complication and be easily used in clinical practice [10]. Ehsani *et al*. reported that rinsing the eyes of patients admitted to the intensive care unit with normal saline did not prevent keratopathy [14]. Bendavid *et al*. showed that using ophthalmic lubricants and contact lenses could limit keratopathy and improve its lesions in patients admitted to special wards [15].

Despite differences in the type of intervention in the above studies, similar results were obtained. According to previous studies, simple eye care protocols such as washing with normal saline should gradually be replaced by more effective interventions such as eye drops and ointments, but still, in many clinical settings, evidence-based interventions are not used. One of the reasons is the weakness of studies in introducing the best and most appropriate eye care solution. In fact, each study ultimately examines one or two interventions. On the other hand, systematic and meta-analysis studies sometimes cannot provide comprehensive results. It is because of the different research communities, sample size, and other variables such as different measurement tools and not considering distorting variables. Therefore, different interventions should be evaluated by researchers in the form of clinical trials.

This study showed that the use of eye care protocol reduced the incidence of corneal ulcers in patients admitted to special wards in the intervention group compared to the control group. In the study of Ehsani *et al*. [14], in addition to daily eye care with normal saline, patients underwent one of three eye care methods, including the use of polyethylene cover, liposic ointment, and artificial tear drops on one eye of each sample for 5 days. As a result, the use of polyethylene rings to cover the eyes was significantly more effective than other methods in maintaining corneal health. Also, all 3 methods of eye care were more effective than washing the eye with normal saline alone [14]. The results of this study are consistent with the present study. Ahmadinejad *et al*. reported that the use of adhesive on patients' eyelids reduced the rate of superficial eye disorders by 20.2%, and in patients who used eye ointment by 3.6%.

As a result, eye ointment was more effective than keeping the eye closed with an adhesive to prevent ocular surface disorders [16], which is consistent with the present study. Koroloff *et al*. stated that polyethylene was more effective in corneal damage than hypromellose [17], which contradicts the results of the present study. It seems that the difference in the type of patients in the ICU is one of the reasons for this contradiction. Alavi *et al*. stated that using moisturizing ointment as a protective method for corneal abrasion could have positive effects in hospitalized patients [9]. This study showed that the use of eye care protocol reduced the incidence of dry eye in patients admitted to special wards in the intervention compared to the control group. In the study of Alavi *et al*., moisturizing ointment as a protective method against dry eye had favorable effects in hospitalized patients [9], corresponding to our results.

Attention to the dry eye has been one of the most important aspects of eye care. Güler *et al*. stated that opening the patient's eyelids increased the incidence of ocular surface disorders [18]. Ahmadinejad *et al*. showed that keeping the eyelids closed without using an eye moisturizer like an ointment was not a complete and effective eye care method [16]. Therefore, this study used eye drops to prevent eye dryness. The present study showed that the use of eye care protocol reduced the incidence of conjunctivitis in hospitalized patients in special wards in the intervention group compared to the control. In this regard, Wilkins *et al*. showed that using hypromellose lubricant was effective in patients with viral conjunctivitis. In this study, short-term use of dexamethasone was more effective than hypromellose in treating acute viral conjunctivitis [19], which is not consistent with our study. In their study, patients had viral conjunctivitis, but in the present study, the criterion for entering the study was the absence of this complication, therefore, the reason for this difference is justifiable, although further studies may be necessary.

In the mentioned studies, positive results were obtained from providing eye care strategies, but comparing these methods in different clinical trial studies is necessary to determine the best eye care method for each group of patients. Some studies used a small sample size, which makes generalization and comparison difficult, so future studies with reasonable sample sizes and comparison of different care methods are needed to provide a standard clinical guideline for nurses and caregivers in the ICU. One of the limitations of this study is the limited duration of the intervention, which suggests that the duration of care interventions be extended to provide a more reliable result within the clinical environment.

## Conclusion

This study showed that using eye care protocol reduced the incidence of keratitis, conjunctivitis, dry eye, and corneal ulcers in patients admitted to special wards. Therefore, this method applied to patients admitted to the intensive care unit can effectively improve eye care and prevent vision impairment after recovery.

## Personal thanks

All the patients and their families, nurses, and doctors of intensive care units of Imam Khomeini and Golestan Hospitals who cooperated with the researchers are thanked and appreciated.
